# Development and validation of sonological classification and scoring system for uterine adenomyosis: A pilot study

**DOI:** 10.12688/f1000research.125700.2

**Published:** 2023-09-20

**Authors:** Sudwita Sinha, Mukta Agarwal, Punam Prasad Bhadani, Ria Roy, Upasna Sinha

**Affiliations:** 1 Obstetrics & Gynecology, AIIMS, PATNA, BIHAR, 801507, India; 2 Pathology, AIIMS, PATNA, Bihar, 801507, India; 3 Community and Family medicine, AIIMS, PATNA, Bihar, 801507, India; 4 Radiology, AIIMS, PATNA, Bihar, 801507, India

**Keywords:** sonological classification of adenomyosis, validation of sonological scoring system, adenomyosis, sonological and pathological agreement, diagnostic accuracy of sonological system of adenomyosis, hysterectomy, heavy menstrual bleeding, dysmenorrhoea

## Abstract

**Background:** Adenomyosis is a common disorder in women of reproductive age. The gold standard for diagnosis is histopathological examination of hysterectomy specimen. However, only a small percentage of women undergo surgery as treatment is primarily hormonal. Non-invasive methods of diagnosis include transvaginal sonography and magnetic resonance imaging. Patient management in adenomyosis is often based on ultrasonographic diagnosis alone, highlighting the importance of a uniform, reproducible, clinically relevant and validated sonological classification and scoring system. Although a few investigators have proposed classification and scoring system for diagnosis of adenomyosis, none of those have been validated yet. This study aimed to propose and validate a new sonological classification and scoring system for adenomyosis.

**Methods:** This was a prospective observational pilot study. A new sonological classification and scoring system of adenomyosis was proposed based on topography, type, size and extent, which was validated by comparing the sonological reporting with histopathological reporting. The main outcome measures that were measured were rate of agreement (Cohen’s kappa) between the findings of sonologist and pathologist; and diagnostic accuracy of the sonological classification of adenomyosis.

**Results:** This pilot study included 30 women who underwent hysterectomy over a time period of one year with ultrasonographic diagnosis of adenomyosis. The rate of agreement (Cohen’s kappa) between the findings of sonologist and pathologist showed substantial agreement (0.703) for topography and almost perfect agreement for type (0.896), extent (0.892) and size (0.898).

**Conclusions:** Our newly proposed sonological classification and scoring system for adenomyosis is valid and can be used for clinical application in interpersonal communication between clinicians, to prognosticate patients about the disease severity, to assess the candidates for surgical management and in further studies to correlate with symptoms severity and effectiveness of medical therapies.

## Introduction

Adenomyosis or ‘cystosarcoma adenoides uterinum’, as initially described by Rokitansky (1860), was defined by Von Recklinghausen (1896) as migration of endometrium glands and stroma into the myometrium. ^1^ Angiogenesis of spiral vessels and smooth muscle hyperplasia and hypertrophy is incited by these migrated endometrium glands leading to junctional zone thickening and diffuse enlargement of the uterus. ^2^ The gold standard for diagnosis is histopathological examination of hysterectomy specimen. ^3^ However, not all women suffering from adenomyosis undergo surgery as the treatment of choice is primarily hormonal – oral progestins, levonorgestrel intrauterine system (LNG-IUS), etc. ^3^ Therefore, tools for non-histologic confirmation of adenomyosis are indubitably required. The non-invasive methods of diagnosis of adenomyosis include transvaginal sonography (TVS) and magnetic resonance imaging (MRI). ^3^ Ultrasonography (USG) is readily available, relatively inexpensive, has no contraindications, and requires no preparation, making it the imaging modality of choice. ^3^ Currently there are no guidelines on USG based classification and scoring system of adenomyosis.

Patient management in adenomyosis is often based on USG diagnosis alone, highlighting the importance of a uniform, reproducible, clinically relevant and validated scoring system for USG findings. Many new sonological classifications of adenomyosis have been proposed in the recent years. ^4^^–^^6^ However, it is necessary to validate such tools so that it can be used in clinical practice. After extensive literature research, we found that to date none of the proposed classification and scoring systems for adenomyosis have been validated yet.

A uniform classification and scoring system for USG findings of adenomyosis can be applied in clinical practice only after validation. Classification of type and degree of adenomyosis will be useful in interpersonal communication between clinicians, to prognosticate patients about the severity of the disease, to assess the candidates for management options and in further research studies to correlate with symptoms severity and effectiveness of medical therapies. This study aimed to propose and validate a new classification and scoring system for adenomyosis. The primary objective was to calculate the rate agreement between the findings of sonologist and pathologist. A secondary objective was to determine the diagnostic accuracy of the sonological classification of adenomyosis described using sensitivity, specificity, positive predictive value, negative predictive value, positive likelihood ratio and area under receiver operator curve.

## Methods

This was a single-centered, hospital-based, prospective, observational pilot study conducted in the Departments of Obstetrics and Gynecology, Radiology and Pathology, AIIMS, Patna after ethical approval over a time period of one year from August 2021 to July 2022. Since this was a pilot study, a sample size of 30 was chosen for the study. A new sonological classification and scoring system for adenomyosis was proposed based on topography, type, size and extent. This classification system was validated by comparing the sonological reporting with the histopathological reporting which is the gold standard investigation to confirm the diagnosis of adenomyosis. Patients attending the Gynecology outpatient department of AIIMS, Patna, with symptoms suggestive of adenomyosis – heavy menstrual bleeding, dysmenorrhea, dyspareunia, pelvic pain and infertility – were asked to get a transvaginal sonography done, which was performed by a sonologist with six years of experience in the Department of Radiology. Patients were asked to evacuate the bladder and then were laid down in lithotomy position. After taking proper consent, conventional two-dimensional transvaginal sonography (by ACCUVIX-XG machine) was done using a 5-9 Megahertz (MHz) transducer. Firstly, the pelvis was assessed to rule out any obvious pathology. Then the uterus was visualized and focused in the transverse and longitudinal planes. Myometrium was examined to look for the presence of any abnormalities. Color doppler analysis was done. Identical ultrasound settings, both grayscale and doppler were set for all patients. Diagnosis of adenomyosis was made when any of the recognized ultrasound feature of adenomyosis was observed – that is minimum number of MUSA features was considered for sonological diagnosis of adenomyosis (2015 MUSA group consensus – the international morphological uterus sonographic assessment). ^3^ MUSA features of adenomyosis includes enlarged globular uterus, asymmetrical thickening of myometrium, myometrial cysts, echogenic sub-endometrial lines and buds, hyperechogenic islands, fan shaped shadowing, irregular or interrupted junctional zones and trans-lesional vascularity on color doppler study. ^3^ Those with confirmed diagnosis of adenomyosis based on USG were classified into different types according to the proposed classification system and a score was given based on the type, size and extent. Their data (including their contact information) was recorded in a register. Patients were counselled regarding different management options. Among these patients, those opting for hysterectomy were included in the study.

Inclusion criteria included premenopausal women (defined by having had menstruation in the last six months), in the age group of 18–52 years, on no hormonal treatment for the past three months, who gave written informed consent, had hysterectomy planned with a USG based provisional diagnosis of adenomyosis and in whom hysterectomy did not require morcellation (uterus was allowed to be divided into two to three pieces, given that the orientation of the specimen was still possible for the pathologist). Exclusion criteria were postmenopausal women or women with no menstrual bleeding for the last six months, ongoing pregnancy, reproductive tract cancer, fibroid uterus, use of gonadotropin-releasing hormone (GnRH) agonist or antagonist or oral progestins or LNG-IUS within last three months prior to USG evaluation and need for morcellation of the uterus.

The patients undergoing hysterectomy were admitted in the Gynecology ward two days prior to surgery. A detailed history including chief complaints, history of presenting illness, past medical or surgical history, menstrual history, obstetric history, family history, drug and allergy history and associated co-morbidities were taken from all patients. General examination, systemic examination and pelvic examination was done and findings were noted. Hysterectomy was performed after proper pre-anesthetic check-up, pre-operative investigations, negative Pap smear and normal endometrial biopsy. Hysterectomy specimens were sent for histopathological examination in the Department of Pathology to confirm the diagnosis, type, topography, size, extent and scoring of adenomyosis (observed on USG) by a pathologist, who was blinded to the findings and score of the sonologist. Data regarding patient’s history, examination findings, associated co-morbidities, investigation results, sonological classification and soring, and peri-operative management were collected from the patients’ file records while they were admitted in the Gynecology ward. Histopathological reporting, classification and scoring data were collected from the Department of Pathology using patients’ clinical reference number.

### Reporting adenomyosis

Adenomyosis on USG was reported as follows:

1. Topography: anterior, posterior, left lateral, right lateral or fundal.
2. Type: on a sagittal section through the uterus where the adenomyotic lesion appeared to be at its largest – localized if >25% of the circumference of the lesion was surrounded by normal myometrium, diffuse if <25% of the circumference of the lesion was surrounded by normal myometrium, adenomyoma when focal adenomyosis was demarcated distinctly and surrounded by hypertrophic myometrium, and mixed if features of both localized and diffuse adenomyosis were seen.
3. Extent: assessed subjectively based on proportion of affected uterine corpus – superficial if <25% was affected, deep if >25% was affected but serosa was not involved and full thickness if entire myometrial thickness was affected.
4. Size of lesion: the largest diameter of the largest lesion was measured and in case of diffuse lesion, uterine size was measured.

Table 1 shows the proposed LAD (localized, adenomyoma, diffuse) classification and scoring system for adenomyosis. Minimum possible score is 3 and maximum possible score is 10.

**Table 1.** LAD (localized, adenomyoma, diffuse) classification and scoring system of adenomyosis. Minimum score-3, maximum score-10.

Topography	Type-score	Extent-score	Size-score
A- anterior	I. Localised-1	a. Superficial-1	1) <2 cm=1
P-posterior	II. Adenomyoma-2	b. Deep-2	2) 2–5 cm=2
LL-left lateral	III. Diffuse-3	c. Full thickness-3	3) >5 cm=3
RL-right lateral	IV. Mixed-4		
F- fundal			

The outcome measures that were estimated included rate agreement between the findings of sonologist and pathologist and diagnostic accuracy of the sonological classification of adenomyosis described using sensitivity, specificity, positive predictive value, negative predictive value, positive likelihood ratio and area under receiver operator curve.

### Statistical analysis

Data was collected offline, entered into Microsoft Excel version 16 and analyzed on SPSS v25 (Statistical package for the social sciences version 25). Cohen’s kappa was calculated by entering the number of agreements in each score. The diagnostic accuracy parameters were estimated by calculating the area under the receiver operator characteristic curve, along with finding the optimum sensitivity and specificity through Youden’s index (sensitivity + specificity -1). The predictive values and likelihood ratios were subsequently calculated taking prevalence of adenomyosis as 23.5%, according to a study done in India by Bupathy Arunachalam and Jayasree Manivasakan. ^7^

### Ethical considerations

The study was conducted after approval of the ethics committee of the institute (IEC-AIIMS-P ALL INDIA INSTITUTE OF MEDICAL SCIENCES, PATNA PHULWARISHARIF PATNA BIHAR-2021/761), dated 27 ^th^ July 2021. Written and informed consent were taken from all participants.

## Results

A total of 30 women were included in the study who underwent hysterectomy with a diagnosis of adenomyosis based on USG. ^8^ The age distribution was as follows: three women were less than 40 years, 13 women were between 40–45 years, 10 women were between 46–50 years and four women were above 50 years of age. All patients were multiparous except for one nulliparous patient. All the patients had a history of heavy menstrual bleeding (HMB) with varying duration of symptom; four patients had HMB for less than a year, 17 patients had HMB for 1–5 years, six patients had HMB for 5–10 years and three patients had HMB for more than 10 years. It was observed that 11 out of 30 patients had symptoms of dysmenorrhea of which three patients had symptoms for more than 10 years, two patients had symptoms for 5–10 years, five patients had symptoms for 1–5 years and one patient had symptoms for less than a year. Only one patient had the symptom of dyspareunia. Only one patient had infertility. Chronic pelvic pain was seen in 14 patients of which two patients had symptoms for more than 10 years, two patients had symptoms for 5–10 years, six patients had symptoms for 1–5 years and four patients had symptom for less than a year. Pelvic organ prolapse was an accompanying symptom seen in four out of 30 patients. The medical co-morbidities associated with adenomyosis included hypothyroidism (10 out of 30 patients), hypertension (eight out of 30 patients) and diabetes mellitus (three out of 30 patients). A history of prior treatment with hormonal therapy was given by 11 out of 30 patients. Anemia was present in 16 patients with pre-operative hemoglobin less than 10 g/dl (grams per deciliter) of which eight required peri-operative blood transfusion. The surgeries that were done in these patients included total abdominal hysterectomy in six patients, total laparoscopic hysterectomy in 20 patients, vaginal hysterectomy with pelvic floor repair in two patients and total laparoscopic hysterectomy with pelvic floor repair in two patients. On histopathology, diagnosis of adenomyosis was confirmed for all patients except for one (29 out of 30 patients). The topographic distribution showed 13 patients with adenomyosis in the anterior wall of the uterus, 10 patients with adenomyosis in the posterior wall of the uterus, five patients with adenomyosis in the fundus of the uterus, three in the right lateral wall and two in the left lateral wall of the uterus. On classifying the type of adenomyosis, localized adenomyosis was seen in 15 patients, diffuse adenomyosis in nine patients and adenomyoma in five patients. Coming to the extent distribution in patients – 16 patients had superficial adenomyosis, seven patients had deep adenomyosis and six patients had full thickness adenomyosis. The size of the largest lesion of adenomyosis was less than 2 cm in 14 patients, 2–5 cm in eight patients and more than 5 cm in seven patients. The total score showed a score less than or equal to 3 in 12 patients, a score between 4–6 in eight patients and a score more than or equal to 7 in nine patients.

The sonographic scoring system had an overall visible agreement with the histopathological scoring system and findings of the pathologist. In Table 2, the agreement was determined by Cohen’s kappa. The kappa value for topography of adenomyosis is 0.703, indicating substantial agreement (0.61–0.80). The sonographic findings of the type, extent and size of adenomyosis had almost perfect agreement to the histopathological findings, as shown by the kappa value of 0.896, 0.892 and 0.898 respectively.

**Table 2.** Rate of agreement between findings of sonologist and pathologist (n=30).

Characteristics of adenomyosis	Cohen’s kappa	Interpretation
Topography	0.703 (0.549–0.856)	Substantial agreement
Type	0.896 (0.760–1.000)	Almost perfect agreement
Extent	0.892 (0.746–1.000)	Almost perfect agreement
Size	0.898 (0.761–1.000)	Almost perfect agreement

Table 3 shows the diagnostic accuracy characteristics of the proposed sonographic scoring system which diagnosed adenomyosis as being present in the participants. All the parameters had 100% sensitivity, and the extent of adenomyosis had the highest specificity of 69.6% and highest positive predictive value of 50%. There was only one participant who did not have any adenomyosis according to histopathology, but came highly positive on sonography, resulting in the test being non-specific for topography. Except for topography, every other parameter can be successfully determined by sonography, as shown by the area under receiver operator characteristic curve being 0.600 and above.

**Table 3.** Diagnostic accuracy of the proposed sonological classification of type of adenomyosis (n=30).

Diagnostic accuracy indicators	Topography	Type	Extent	Size
Sensitivity (%)	100	100	100	100
Specificity (%)	0	60	69.6	63.6
Positive predictive value (%)	23.5	43.8	50.0	46.0
Negative predictive value (%)	0.0	0.0	0.0	0.0
Positive likelihood ratio	1	2.5	3.3	2.75
Area under receiver operator characteristic curve	0.0	0.600	0.696	0.636

## Discussion

The above study shows that most of the patients with adenomyosis who landed up in surgery (hysterectomy) belonged to the age group of 40–50 years. This may be due to the fact that medical and hormonal treatments are tried first in younger age groups and surgery is done only when hormonal therapy fails. All the patients undergoing surgery were multiparous except for one nulliparous patient who was so much affected by the symptoms that desire of fertility was not her first priority, and she was ready to go for adoption. The reason behind maximum patients undergoing surgery being multiparous can also be explained by the conservative attitude towards treatment in nulliparous women so as to conserve the uterus. The most common symptoms seen in adenomyosis patients undergoing hysterectomy were heavy menstrual bleeding (100%), chronic pelvic pain (46.67%) and dysmenorrhea (36.67%). Heavy menstrual bleeding resulted in anemia in many patients as is shown by the fact that 53.33% of patients had hemoglobin less than 10 g/dl before surgery that necessitated blood transfusion in half of these patients (26.67%). Only one patient had infertility as we generally don’t do hysterectomy in an infertile patient. This patient was however so much burdened with the disease symptoms that she wanted complete surgery to relieve her symptoms despite counselling against surgery and offering conservative management options. It is also seen that dyspareunia was complained by only one out of 30 patients. This might be underreporting of this symptom due to several factors in this part of the country with the majority of women belonging to lower socio-economic status. Some of the factors may include considering it a taboo, stigma or shameful topic, shyness, embarrassment, reluctance to raise the topic of sexual problems with clinicians, patriarchal dominance where females don’t have any say against sexual discomfort, etc. Pelvic organ prolapse was seen in four patients which may be unrelated to the etio-pathogenesis of adenomyosis. The most common associated medical co-morbidities were hypothyroidism (33.33%), hypertension (26.67%) and diabetes mellitus (10%). These factors may have also contributed to heavy menstrual bleeding independently. In 11 out of 30 patients, prior treatment with hormonal therapy had failed which resulted in patients opting for surgery for relief of symptoms. On histopathology, diagnosis of adenomyosis was confirmed in 29 out of 30 patients (96.67%). The topographic distribution showed 43.33% anterior wall adenomyosis, 33.33% posterior wall adenomyosis, 16.67% fundal adenomyosis, 10% right lateral wall and 6.67% left lateral wall adenomyosis. Thus, it can be concluded that adenomyosis is more common in fundo-anterior and posterior regions of the uterus. On classifying the type of adenomyosis, the most common type seen was localized adenomyosis (50%), followed by diffuse adenomyosis (30%) and adenomyoma (16.67%). Adenomyoma was the least common type of adenomyosis seen. More than half (53.33%) of patients had superficial adenomyosis, 23.33% of patients had deep adenomyosis and 20% of patients had full thickness adenomyosis. Majority of the patients had small size adenomyosis (46.67%), whereas adenomyosis of more than 5 cm was seen in 23.33% of patients. The total score showed a score less than or equal to 3 (mild) in 40%, a score between 4–6 (moderate) in 26.67% and a score more than or equal to 7 (severe) in 30% of patients.

The sonographic scoring system had an overall visible agreement with the histopathological scoring system and findings of the pathologist with topography of adenomyosis having substantial agreement whereas type, extent and size of adenomyosis having almost perfect agreement to the histopathological findings. This suggests that our proposed classification and scoring system for adenomyosis is valid and can be used in clinical practice. The clinical relevance will be particularly more in infertility patients where hysterectomy is not being considered and an accurate diagnosis of adenomyosis can help fertility specialists to plan treatment appropriately or counsel and prognosticate such patients.

Among the diagnostic accuracy characteristics of the proposed sonographic scoring system which diagnosed adenomyosis as being present in the participant, sensitivity was 100% for all parameters and specificity was highest for extent of adenomyosis (69.6%). The highest positive predictive value of 50% was also seen for extent of adenomyosis. However, there was only one participant who did not have any adenomyosis according to histopathology, but came highly positive on sonography, resulting in the test being non-specific for topography. Except for topography, every other parameter can be successfully determined by sonography, as shown by the area under receiver operator characteristic curve being 0.600 and above.

### Limitations

The limitations of this study include its small sample size and unavailability of three-dimensional (3-D) ultrasound machines for transvaginal sonography which could have given more accurate imaging diagnosis. Hence, there is a need to continue this study with larger group of patients to revalidate this scoring system before it can be used in clinical practice.

## Conclusion

To conclude, the present study successfully proposed and validated a sonological classification and scoring system for adenomyosis which can be applied in clinical practice for interpersonal communication between clinicians, further research studies correlating disease severity with severity of clinical symptoms, to prognosticate patients about the severity of their disease, deciding the different management options for patients and following up patients for effectiveness of medical and hormonal therapies; which would not be possible if using MUSA classification as there is no scoring system in its classification. Further studies with larger sample size are still required to correlate disease severity according to score with the severity of clinical symptoms.

## Data availability

### Underlying data

Figshare: adenomyosis data file 1. https://doi.org/10.6084/m9.figshare.20708965. ^8^

This project contains the following underlying data:

• adenomyosis data file 1.csv (data mentioning the age, parity, symptoms, co-morbidities, sonological and histopathological classification and scoring and the surgeries performed)

Data are available under the terms of the Creative Commons Attribution 4.0 International license (CC-BY 4.0).
