# Supplementary material for: Development and validation of sonological classification and scoring system for uterine adenomyosis: A pilot study
Source: F1000Res. 2023 Sep 20;11:1138. Originally published 2022 Oct 5. [Version 2] doi: 10.12688/f1000research.125700.2 (PMC10587656; doi:10.12688/f1000research.125700.2)
Supplement: Peer review history

# Reviewer report: Reviewer response for version 2

**DOI:** 10.5256/f1000research.156254.r208139

**Published:** 2023-10-19

**Authors:** Maria Grazia Porpora

**Affiliations:** 1 Department of Maternal and Child Health and Urological Sciences, Policlinico Umberto I, Sapienza University of Rome, Rome, Italy

Authors made the required change, therefore the manuscript is now suitable for publication.

Is the work clearly and accurately presented and does it cite the current literature?

Yes

If applicable, is the statistical analysis and its interpretation appropriate?

Yes

Are all the source data underlying the results available to ensure full reproducibility?

Yes

Is the study design appropriate and is the work technically sound?

Yes

Are the conclusions drawn adequately supported by the results?

Yes

Are sufficient details of methods and analysis provided to allow replication by others?

Yes

Reviewer Expertise:

Endometriosis, minimally invasive gynecology

I confirm that I have read this submission and believe that I have an appropriate level of expertise to confirm that it is of an acceptable scientific standard.

# Reviewer report: Reviewer response for version 1

**DOI:** 10.5256/f1000research.138035.r168055

**Published:** 2023-04-12

**Authors:** Maria Grazia Porpora

**Affiliations:** 1 Department of Maternal and Child Health and Urological Sciences, Policlinico Umberto I, Sapienza University of Rome, Rome, Italy

Thank you for inviting me to review the manuscript entitled “Development and validation of sonological classification and scoring system for uterine adenomyosis: A pilot study” by Sudwita Sinha et al.

 The aim of this pilot study is to assess a valuable sonological classification and scoring system able to better address the diagnosis of uterine adenomyosis.

 The Abstract is clear and accurately summarizes the contents of the paper. Methods are well explained. The Results are consistent with the objectives of the study. References are updated. English language is clear and easily understood.

 The greatest limitation of the study, as reported by Authors, is the low sample size. Authors should emphasize on the need of a larger group of patients to validate this study model.

 Moreover, as the well-known MUSA criteria are the most suitable accepted classification system for adenomyosis, Authors should clarify why and how their sonological classification and scoring system is ought to be more useful and adoptable by experts in this field.

 Therefore, the article will be suitable for indexing in F1000 Research after minor revision.

Is the work clearly and accurately presented and does it cite the current literature?

Yes

If applicable, is the statistical analysis and its interpretation appropriate?

Yes

Are all the source data underlying the results available to ensure full reproducibility?

Yes

Is the study design appropriate and is the work technically sound?

Yes

Are the conclusions drawn adequately supported by the results?

Yes

Are sufficient details of methods and analysis provided to allow replication by others?

Yes

Reviewer Expertise:

Endometriosis, minimally invasive gynecology

I confirm that I have read this submission and believe that I have an appropriate level of expertise to confirm that it is of an acceptable scientific standard, however I have significant reservations, as outlined above.

# Reviewer report: Reviewer response for version 1

**DOI:** 10.5256/f1000research.138035.r153725

**Published:** 2022-11-07

**Authors:** Rooma Sinha

**Affiliations:** 1 Departments of Gynaecology, Apollo Hospitals, Hyderabad, Telangana, India; 2 Macquarie University, Sydney, Australia

The study is well designed and written and can be used in clinical setting to confirm the diagnosis of adenomyosis.

 It's clinical relevance can be more in infertility patients where hysterectomy is not being considered and an accurate diagnosis of adenomyosis can help fertility specialists to plan treatment appropriately or counsel and prognosticate such patients. This point can be included in the discussion. 

 Although this study attempts to validate this scoring system, the number of participants is small. Perhaps the authors can continue this study in future and revalidate it. This at present can be considered as a pilot study. 

 Did the authors take all the characters described in MUSA into consideration for sonological diagnosis of adenomyosis or a minimum number. Could the authors clarify this as all features described are not seen in all patients. This would be important for diagnosis in clinical setting.

Is the work clearly and accurately presented and does it cite the current literature?

Yes

If applicable, is the statistical analysis and its interpretation appropriate?

Yes

Are all the source data underlying the results available to ensure full reproducibility?

Yes

Is the study design appropriate and is the work technically sound?

Yes

Are the conclusions drawn adequately supported by the results?

Yes

Are sufficient details of methods and analysis provided to allow replication by others?

Partly

Reviewer Expertise:

Minimally invasive Gynecology

I confirm that I have read this submission and believe that I have an appropriate level of expertise to confirm that it is of an acceptable scientific standard.
